# Nanocomposites of Nitrogen-Doped Graphene Oxide and Manganese Oxide for Photodynamic Therapy and Magnetic Resonance Imaging

**DOI:** 10.3390/ijms232315087

**Published:** 2022-12-01

**Authors:** Haseeb A. Khan, Yong-Kyu Lee, Mohammed Rafi Shaik, Sara T. Alrashood, Aishah A. Ekhzaimy

**Affiliations:** 1 Department of Biochemistry, College of Science, King Saud University, Riyadh 11451, Saudi Arabia; 2 Department of Chemical and Biological Engineering, Korea National University of Transportation, Chungju, Republic of Korea; 3 Department of Chemistry, College of Science, King Saud University, Riyadh 11451, Saudi Arabia; 4 Department of Pharmaceutical Chemistry, College of Pharmacy, King Saud University, Riyadh 11451, Saudi Arabia; 5 Division of Endocrinology, Department of Medicine, King Khalid University Hospital, Riyadh 11472, Saudi Arabia

**Keywords:** nanoparticles, manganese oxide, graphene oxide, MRI, photodynamic therapy

## Abstract

Cancer is a leading cause of death worldwide. Conventional methods of cancer treatment, including chemotherapy and radiotherapy, are associated with multiple side effects. Recently, photodynamic therapy (PDT) has emerged as an effective therapeutic modality for cancer treatment without adversely affecting normal tissue. In this study, we synthesized nitrogen doped graphene (NDG) and conjugated it with Mn_3_O_4_ nanoparticles to produce NDG-Mn_3_O_4_ nanocomposite with the aim of testing its bimodal performance including PDT and magnetic resonance imaging (MRI). We did not use any linker or binder for conjugation between NDG and Mn_3_O_4_, rather they were anchored by a milling process. The results of cell viability analysis showed that NDG-Mn_3_O_4_ nanocomposites caused significant cell death under laser irradiation, while control and Mn_3_O_4_ nanoparticles showed negligible cell death. We observed increased generation of singlet oxygen after exposure of NDG-Mn_3_O_4_ nanocomposites, which was directly proportional to the duration of laser irradiation. The results of MRI showed concentration dependent enhancement of signal intensity with an increasing concentration of NDG-Mn_3_O_4_ nanocomposites. In conclusion, NDG-Mn_3_O_4_ nanocomposites did not cause any cytotoxicity under physiological conditions. However, they produced significant and dose-dependent cytotoxicity in cancer cells after laser irradiation. NDG-Mn_3_O_4_ nanocomposites also exhibited concentration-dependent MRI contrast property, suggesting their possible application for cancer imaging. Further studies are warranted to test the theranostic potential of NDG-Mn_3_O_4_ nanocomposites using animal models of cancer.

## 1. Introduction

Cancer is one of the deadliest and costliest diseases and is the second leading cause of death worldwide. Photodynamic therapy (PDT) is a promising treatment modality for cancer with minimal side effects and is expected to replace traditional chemotherapy, which is associated with numerous adverse effects. PDT involves combination of light and a photosensitizer (PS), which is activated by absorption of light of a specific wavelength, causing the generation of potentially toxic reactive oxygen species (ROS) that induce a cascade of intracellular molecular events resulting in targeted tissue damage [1,2]. Sun et al. have reviewed the application of metal-based nanoparticles (NPs) for PDT of cancer [3]. Metal oxide-based nanomaterials have also significantly impacted the landscape of healthcare, including in the areas of diagnosis and therapeutic applications [4]. Metal oxides have demonstrated great potential in PDT and magnetic resonance imaging (MRI) in diagnostic radiology. A majority of the transition metals-based oxides offer several advantages in the field of biomedicines due to their biocompatibility and non-toxicity [5]. Even some of them, such as iron oxide, have been approved as an MRI contrast agent by concerned authorities [6]. Therefore, metal oxide NPs have the potential to serve as both therapeutic and imaging agents, particularly, manganese oxide (Mn_3_O_4_) nanoparticles are considered as effective in tumor diagnosis and treatment due to their decent biocompatibility, in-vivo imaging performance and tumor microenvironment (TME) responsiveness [7]. Notably, Mn_3_O_4_ is consists of Mn^2+^ and Mn^3+^, due to which it is extremely sensitive to the redox environment in the cell and rapidly decomposes upon exposure to glutathione (GSH) [8]. Tumor specific antibodies functionalized Mn_3_O_4_ NPs were applied as T1 MRI contrast agent for selective imaging of cancer cells [9]. To avoid some limitations, such as aggregation, poor water dispersibility, high dermal toxicity and low clearance of these NPs, several stabilizing ligands have applied to the surfaces of NPs which make them stable and suitable for therapeutic applications [10]. For instance, folic acid (FA) has been used as ligand for targeting folate receptors (FR), a tumor-associated protein over-expressed in cancer cells having high binding affinity toward folic acid [11]. Recently, transitional metal oxide NPs including Mn_3_O_4_, have been effectively combined with a variety of 2D materials, specially graphene, which has received promising attention for phototherapy due to its excellent photosensitizer properties [12]. Graphene is made up of a single layer of carbon atoms arranged in a honeycomb structure, demonstrating specific combination of physiochemical properties, such as, high surface area (2630 m^2^ g^−1^), optimal thermal conductivity (~5000 Wm K^−1^), and remarkable optical transparency, which make it excellent candidate for drug delivery and therapeutic applications [13]. However, its hydrophobicity causes irreversible agglomeration, which is a great obstacle for utilizing its drug career properties [14]. On the other hand, the oxidation of graphene into graphene oxide (GO) significantly reduces its aggregation tendency [15]. GO exhibits amphiphilic nature due to the presence of hydrophobic graphene moiety and hydrophilic edges; the former property is important for carrying water-insoluble drugs through non-covalent bonding, π-π stacking or hydrophobic interaction or hydrogen bonding [16] whereas the latter property not only provides anchor sites for functionalization [14,17] but also maintains colloidal stability due to negative surface charge [18]. When dispersed in water, GO attains a negative surface charge due to ionization of hydroxyl and carboxylic groups. The magnitude of this negative charge is sufficient to cause electrostatic repulsion resulting in stable dispersion of GO in water [18]. 

The water dispersibility of GO is considered better than the water dispersibility of carbon nanotubes (CNTs) [19]. However, GO contains a variety of oxygen containing random functional groups, which inhibit the homogeneous binding of the NPs on its surface. Therefore, to increase the significant amounts of active sites on the surface of GO, the GO is doped with nitrogen which may provide homogeneous nucleation sites [20]. Nitrogen-doped GO is easily dispersed in solutions, that allows it to be used in higher concentrations. Nitrogen doping not only improves the stability of GO but also enhances its optical and catalytic properties [21,22]. Moreover, compared to other carbon-based nanomaterials, GO offers additional advantages such as cost effective [15,23], large surface area for drug binding and fewer toxic metallic impurities [19]. Biological investigations of GO, both in-vitro and in-vivo have no consensus results and sometimes the results are in contradiction [24]. 

We hypothesized to utilize the PDT property of GO [25,26,27] and MRI contrast property of manganese oxide (Mn_3_O_4_) [28,29] for developing a bimodal nanocomposite with therapeutic as well as diagnostic abilities. We therefore synthesized nitrogen-doped graphene (NDG) and conjugated it with Mn_3_O_4_ nanoparticles to produce NDG-Mn_3_O_4_ nanocomposite with the aim of testing its bimodal performance including PDT and MRI. We studied the cytotoxicity of these nanocomposites and evaluated their efficiency in PDT and MRI using in-vitro models. We also tested the ability of NDG-Mn_3_O_4_ nanocomposites to generate ROS under laser irradiation as a potential mechanism of their toxicity in cancer cells. This is probably the first study reporting the nanocomposite of NDG and Mn_3_O_4_ nanoparticles. 

## 2. Results

**Figure 1:**
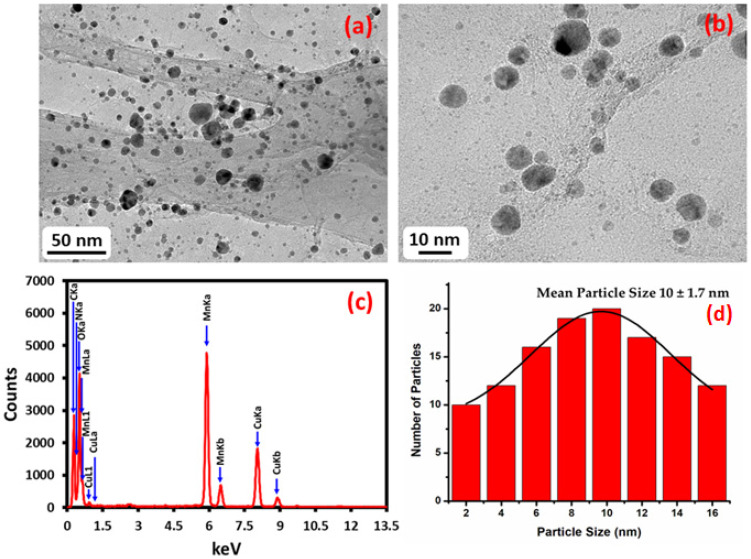
High-resolution transmission electron microscopy (HRTEM) images of the NDG-Mn_3_O_4_ nanocomposite (**a**) low magnification image, (**b**) magnified image, (**c**) energy-dispersive X-ray spectroscopy of NDG-Mn_3_O_4_ nanocomposite and (**d**) particle size distribution of NDG-Mn_3_O_4_ nanocomposite.

The results of high resolution transmission electron microscopy (HRTEM) displayed the existence of spherical shaped Mn_3_O_4_ nanoparticles on the surface of NDG within the range of 5–15 nm (Figure 1). The Mn_3_O_4_ NPs are well distributed on the surface of NDG as the magnified image indicates the shape and crystallinity of these NPs. The Mn_3_O_4_ NPs are not bonded covalently but are held by physisorption on the NDG surface by Vander Waals interactions. The elemental composition of NDG-Mn_3_O_4_ nanocomposite, analyzed by energy-dispersive X-ray spectroscopy, showed intense signals at 0.65, 5.88, and 6.65 keV strongly suggesting that ‘Mn’ was the major element, which has an optical absorption in this range owing to the surface plasmon resonance (SPR). Other signals that were found in the range of 0.0–0.5 keV signified the absorption of carbon, nitrogen and oxygen, confirming the formation of NDG-Mn_3_O_4_ nanocomposite. The average particle size of the NDG-Mn_3_O_4_ nanocomposite was found to be 10 ± 1.7 nm (Figure 1).

**Figure 2:**
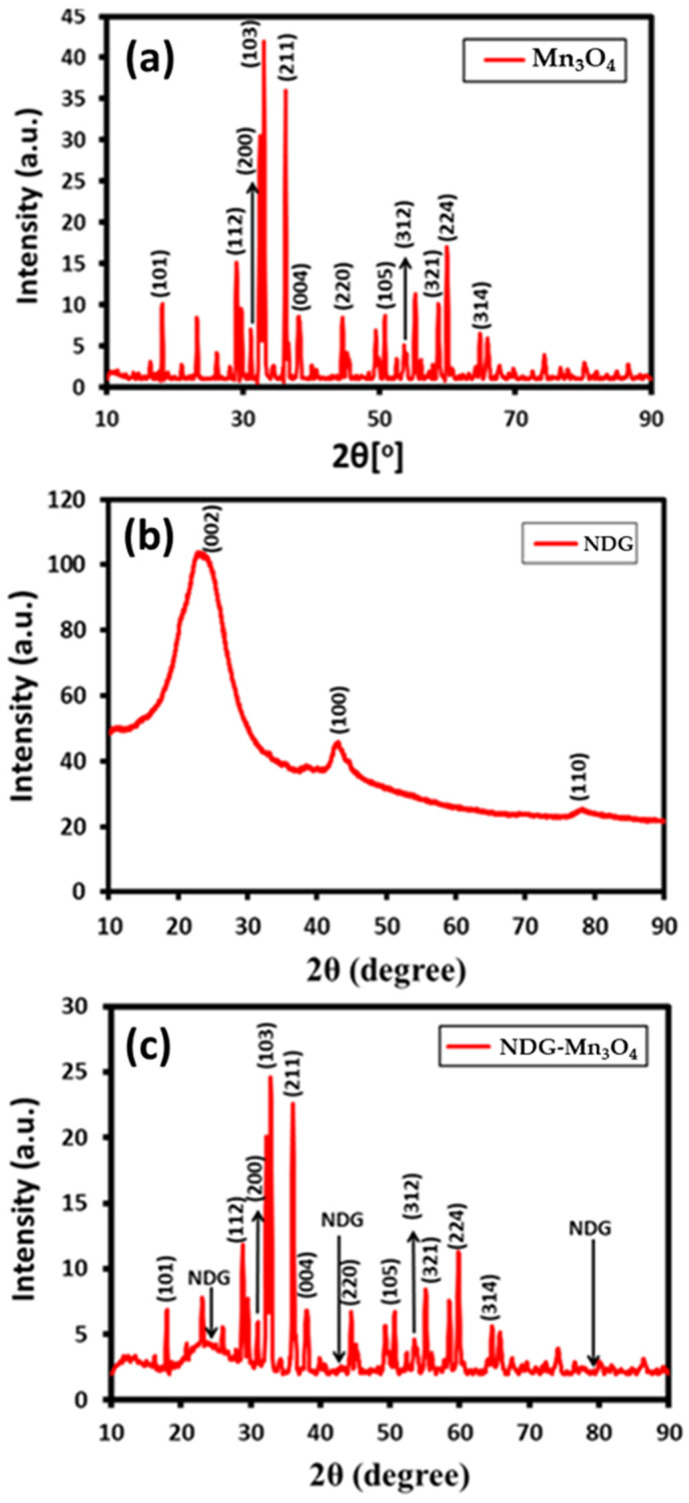
XRD pattern of (**a**) Mn_3_O_4_ nanoparticles, (**b**) NDG and (**c**) NDG-Mn_3_O_4_ nanocomposite.

The XRD pattern of Mn_3_O_4_ NPs shown in Figure 2a exhibits characteristics peaks at 18.2° (101), 29.1° (112), 31.2° (200), 32.5° (103), 36.3° (211), 38.2° (004), 44.6° (220), 50.8° (105), 53.8° (312), 58.7° (321), 60.0° (224), and 64.8° (314), which points to the formation of manganese oxide NPs (Figure 2a) These peaks reveal that the as-obtained Mn_3_O_4_ NPs exist in single phase hexagonal wurtzite structure, besides, the data clearly matched with the standard Mn_3_O_4_ phase reported in the literature (JCPDS Card No. 24-0734) [30]. Notably, the sharp diffraction peaks point toward the highly crystalline and well-disperse nature of nanoparticles which clearly matched with the Hausmannite crystal phase [31]. On the other hand, the XRD pattern of NDG-Mn_3_O_4_ nanocomposite showed the appearance of a broad peak at ~22.4° (002) (Figure 2b) that confirmed the reduction of graphene oxide and formation of NDG [32]. Furthermore, there is no broadening or shift of the (002) peak, proving that there is no change in the interlayer spacing of graphene after nitrogen-doping. No significant change in the full width at half-maximum (FWHM) of the (002) diffraction peak indicates the similar crystallite size before and after nitrogen doping [33]. In case of the composite, the XRD pattern of which is shown in Figure 2c, characteristic diffraction peaks of both Mn_3_O_4_ and N-doped graphene are present, which clearly indicate the formation of hybrid material.

**Figure 3:**
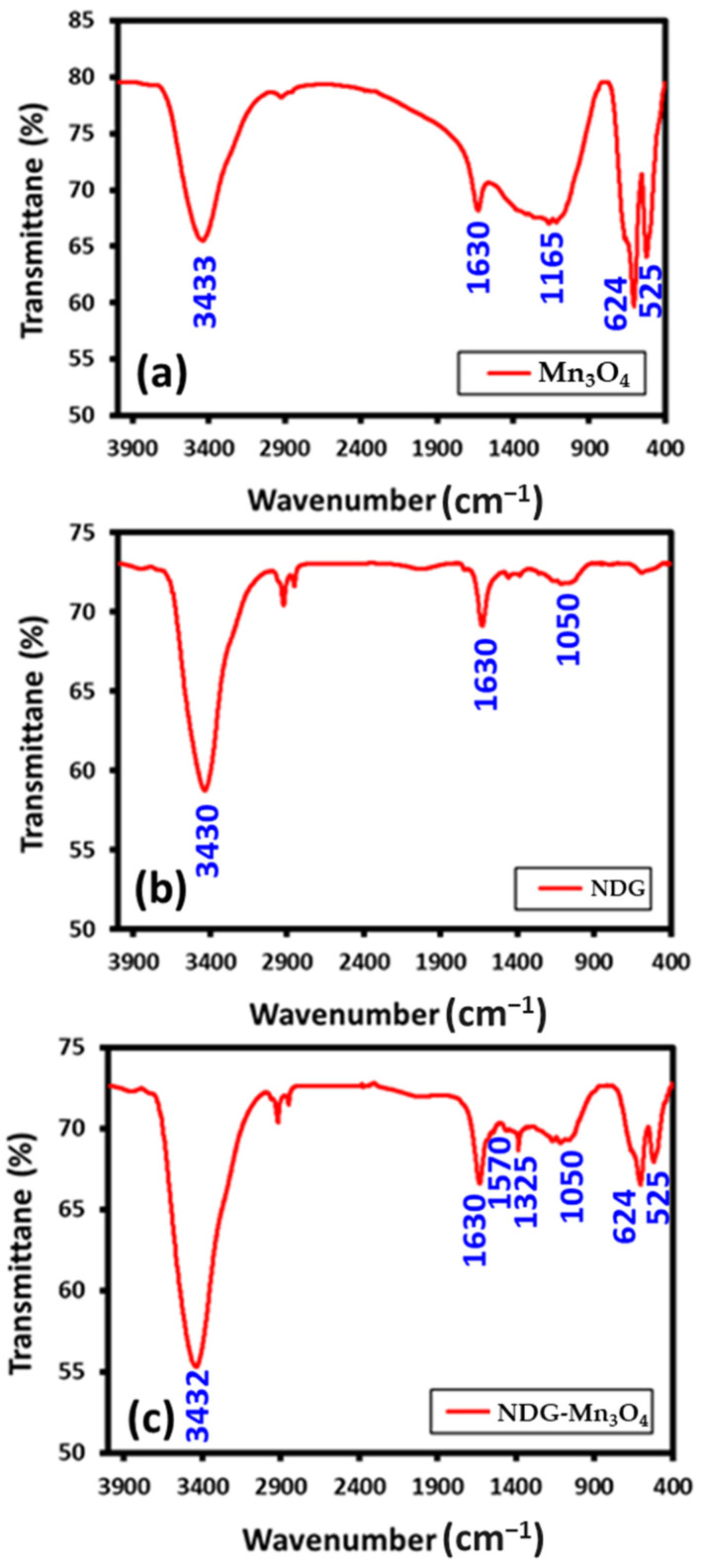
FT-IR spectra of (**a**) Mn_3_O_4_ NPs, (**b**) NDG and (**c**) NDG-Mn_3_O_4_ nanocomposite.

FT-IR spectra of Mn_3_O_4_ NPs displayed the characteristic peak of Mn-O, stretching mode in the range of 624 cm^−1^ while the vibrational frequency associated to the Mn-O distortion vibration poisoned at 525 cm^−1^ (Figure 3a). The characteristic narrow and broad bands located at 3420 and 1600 cm^−1^ were related to the hydroxyl (-OH) groups absorbed by the samples or potassium bromide. FT-IR spectra of NDG are shown in Figure 3b. FT-IR spectra of NDG-Mn_3_O_4_ displayed the graphene oxide intense bands for C=C stretching (~1630 cm^−1^), C–O–C stretching (~1209 cm^−1^), C–O stretching (~1050 cm^−1^). The nitrogen doping in the sample was confirmed by the presence of two characteristic peaks at ~1325 and ~1570 cm^−1^, which were attributed to the stretching of the C–N bond from the secondary aromatic amine, which pointed toward bonding between carbon and nitrogen including the existence of other absorption bands of ‘Mn’ at 624 and 525 cm^−1^ clearly indicating the formation of HRG-Mn_3_O_4_ nanocomposite (Figure 3c).

**Figure 4:**
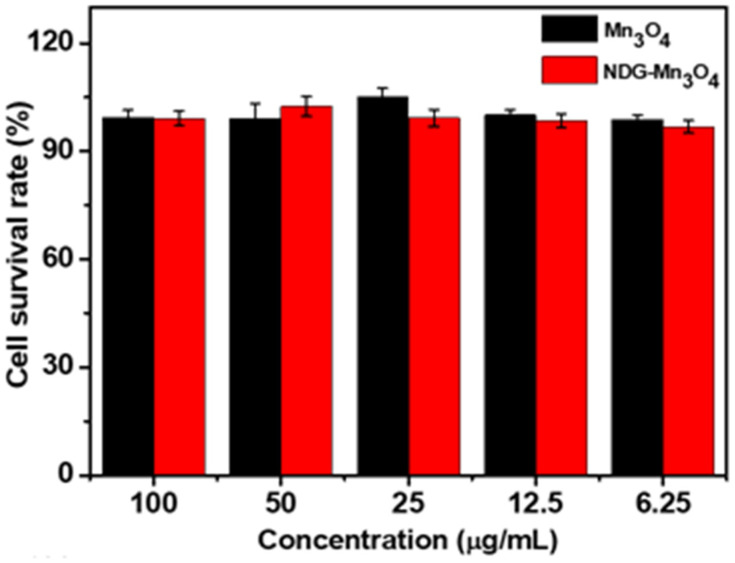
Cytotoxicity analysis showing cell viability of A549 cells treated with different concentrations of Mn_3_O_4_ and NDG-Mn_3_O_4_ nanocomposites. Values are means of three replicates ± standard error.

**Figure 5:**
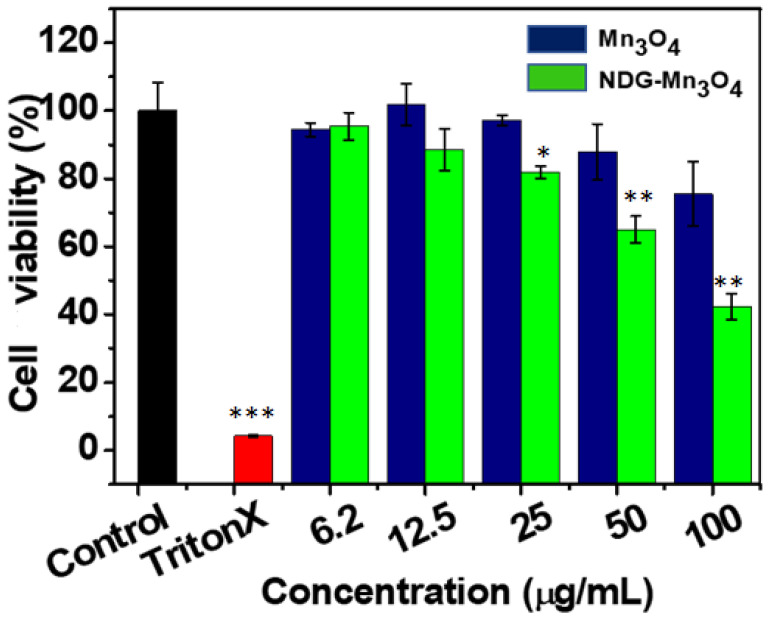
Cell viability of A549 cells incubated with PBS (control), Triton X-100 (negative control) and different concentrations of, Mn_3_O_4_ and NDG-Mn_3_O_4_ nanocomposites in presence of 670 nm laser irradiation (0.1 W/cm^2^) for 5 min. Data is represented as mean values of three replicates (±) standard deviations. * *p* < 0.05, ** *p* < 0.01 and *** *p* < 0.001 versus respective control groups.

The results of cell viability analysis using MTT assay showed that exposure of Mn_3_O_4_ and NDG-Mn_3_O_4_ in the concentration range of 6.25–100 μg/mL did not cause any cytotoxicity (Figure 4). However, NDG-Mn_3_O_4_ nanocomposites displayed significant cells death under laser irradiation for 5 min, while PBS (control) and Mn_3_O_4_ NPs showed negligible cell death (Figure 5). Almost 100% cells were viable when treated with PBS whereas 41% for cancer cells survived after the treatment of 100 µg/mL concentration of NDG-Mn_3_O_4_ nanocomposites along with 5 min of laser irradiation. The effect of NDG-Mn_3_O_4_ nanocomposites on the cytotoxicity of A549 cells was concentration-dependent and only the concentrations of 25 µg/mL and above were found to be effective in killing the cells under laser irradiation (Figure 5).

**Figure 6:**
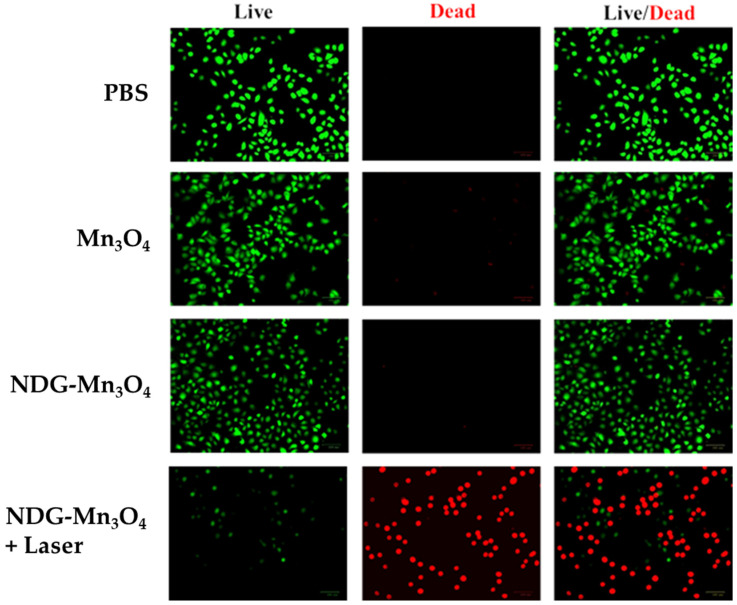
Fluorescence microscopy of A549 cells stained with fluorescein diacetate (green emission for live cells) and propidium iodide (red emission for dead cells) with PBS (control), Mn_3_O_4_ and NDG-Mn_3_O_4_ nanocomposites with/without laser irradiation (670 nm, 0.1W/cm^2^) for 5 min.

The results of in-vitro photodynamic therapy are shown in Figure 6. Without laser irradiation, none of the treatments including PBS, Mn_3_O_4_, or NDG-Mn_3_O_4_ caused any cellular damage as almost all the cells appeared green. After 5 min laser irradiation, NDG-Mn_3_O_4_ nanocomposites killed 68% of the cancer cells (shown as red dots) whereas the treatments of PBS and Mn_3_O_4_ did not cause any significant cellular damage under laser irradiation (Figure 6). 

**Figure 7:**
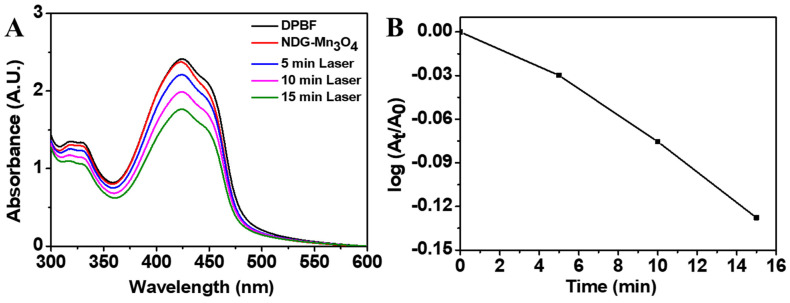
Time-dependent absorption spectra of 1,3-diphenylisobenzofuran (DPBF) in the presence of NDG-Mn_3_O_4_ under laser irradiation (**A**). Absorbance decrease of DPBF at 426 nm at different time points under laser irradiation at room temperature (**B**). (A_t_ = Absorbance at time t, A_0_ = absorbance at time 0).

To evaluate the ^1^O_2_ generation from NDG-Mn_3_O_4_ nanocomposites under laser irradiation, we measured the absorbance of 1,3-diphenylisobenzofuran (DPBF) after laser irradiation (670 nm, 0.1 W/cm^2^) at different time points (Figure 7). The DPBF absorbance decreased with increasing the laser irradiation time, indicating the generation of singlet oxygen from NDG-Mn_3_O_4_ nanocomposites is directly proportional to the duration of laser irradiation (Figure 7). 

**Figure 8:**
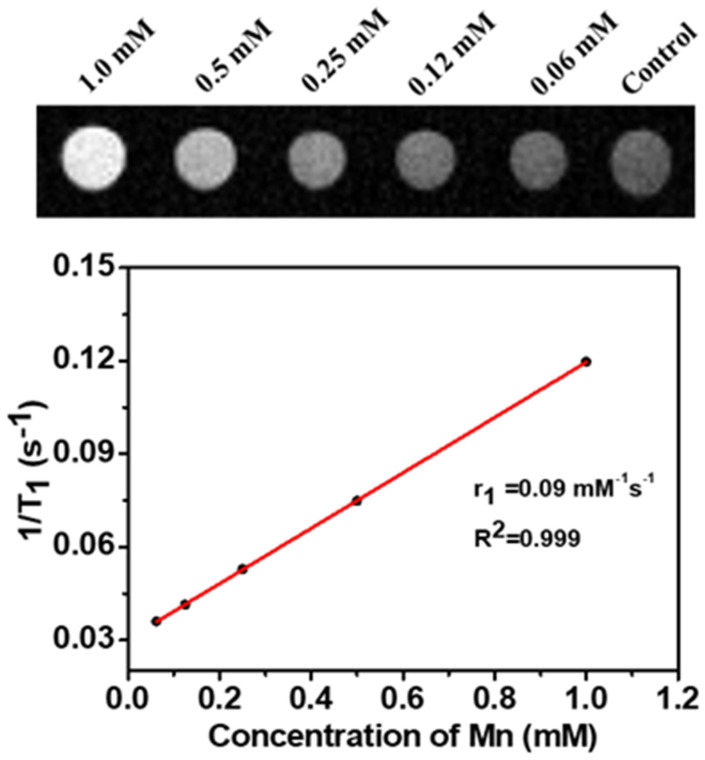
T_1_-weighted MR imaging of NDG-Mn_3_O_4_ nanoparticles in aqueous suspension and the T1 relaxivity plot of aqueous suspension of NDG-Mn_3_O_4_ nanoparticles. The concentration range of 0.06–1.0 mM of Mn is equivalent to approximately 18–152 µg/mL of NDG-Mn_3_O_4_ nanoparticles.

For testing the effectiveness of NDG-Mn_3_O_4_ nanocomposites toward diagnostic standpoint, we investigated whether these nanoparticles have MRI contrast properties or not. Various concentrations of nanoparticles were subjected to imaging by 3T MRI scanner. The result demonstrated a concentration dependent enhancement of signal intensity with increasing concentration of NDG-Mn_3_O_4_ nanocomposites. The r1 value was found to be 0.09 mM^−1^s^−1^ (Figure 8).

## 3. Discussion 

In this study, we conjugated nitrogen doped GO with Mn_3_O_4_ nanoparticles to explore the PDT and MRI potentials of these entities. Nafiujjaman et al., [34] developed a ternary hybrid probe as a dual imaging-guided PDT agent, which consisted of Mn_3_O_4_ and graphene quantum dots (GQD) linked by polydopamine. Conjugation of dextran (DEX) with GO has been shown to remove agglomeration and improve the stability of GO in biological solutions [25]. Kim et al., [35] have shown that DEX coated GO (DEX-GO) nanoparticles are highly biocompatible, maintaining a high cellular viability (>80%) at high concentrations (450 μg/mL). The hybrid DEX-GO nanoparticles also showed significantly higher (18.6 fold) optical density in the NIR region as compared to GO alone, suggesting the application of DEX-GO for PDT [25]. Chen et al., [36] coated iron oxide on DEX-GO nanoparticles and observed that the transformed nanoparticles not only showed better MRI contrast property but also exhibited negligible toxicity.

The results of cell viability analysis showed that NDG-Mn_3_O_4_ nanocomposites did not cause any cytotoxicity unless activated by laser irradiation that resulted in concentration dependent cytotoxicity in lung cancer cells (Figure 5). These biochemical findings were supported by fluorescence microscopy observations, suggesting that NDG-Mn_3_O_4_ nanocomposites initiate cytotoxic properties only under laser irradiation (Figure 6). We used 670 nm laser in order to keep the wavelength within the red optical window (620–750 nm). Visible red radiation is able to activate photosensitizers in deep tumors without causing phototoxicity to normal tissue [37,38]. In our study, NDG-Mn_3_O_4_ nanocomposites killed 68% of cancer cells which is more effective than GQD-PDA-Mn_3_O_4_ nanoparticles (51% cell death) as reported earlier [34]. The mechanism of laser-induced toxicity of NDG-Mn_3_O_4_ nanocomposites can be multifactorial. Nafiujjaman et al. [34] have reported that laser irradiation during PDT triggered the disruption of cellular membranes resulting in a higher cellular uptake of the GQD-PDA-Mn_3_O_4_ nanoparticles compared to graphene quantum dots. This selective transport across the cell membrane might have been influenced by the size, shape and surface chemistry of nanoparticles. 

GO-based nanomaterials possess improved tumor-passive targeting effect and comparatively higher tumor uptake than CNTs due to the enhanced permeability and retention effects, attributed to the peculiar two-dimensional structure and small lateral size of GO [39]. By virtue of its unique optical properties, GO can be used for live cell imaging (due to near infrared photoluminescence) [40,41,42] as well as for PDT (due to free radical generation by optical absorption) [43,44,45]. Because of the minimal cellular autofluorescence in the NIR region, the chances of interference during imaging are also reduced [41]. Thus, intracellular tracking of GO-based nanomaterials can be performed without conjugation with fluorescence dyes, due to the photoluminescence property of GO. 

The mechanism of PDT is complex and not fully understood. It has been suggested that singlet oxygen (^1^O_2_) plays a key role in PDT, which is formed in the molecules of lipids and proteins of cell membranes and intracellular organelles when exposed to the quantum of light [46]. Singlet oxygen is cytotoxic for living cells due to its strong oxidizing property [47]. Our results showed that NDG-Mn_3_O_4_ nanocomposites caused ^1^O_2_ generation under laser irradiation in a time-dependent manner and longer exposure to laser irradiation produced excessive ROS generation (Figure 7). Because uncontrolled generation of ROS is deleterious to normal cells [48], we selected 5 min laser irradiation in further experiments. Sustained elevated levels of ROS leads to irreparable cellular damage through oxidation of nucleic acids, lipids, and proteins, ultimately resulting in cell death through apoptosis or necrosis. There are different scales of oxidative stress ranging from physiological oxidative stress to excessive and toxic oxidative burden [49]. Photosensitizers with near-infrared (NIR) fluorescence as well as efficient ROS generation ability have been used for precise diagnosis and simultaneous treatment of cancer [50]. Liu et al., [51] designed a novel multi-functional nanosystem in which cisplatin was loaded in MnO_2_-doped GO and functionalized with a photosensitizer (Ce6). The nanosystem was equipped with intelligent functions including: (1) decomposition of H_2_O_2_ into oxygen to relieve the tumor hypoxia; (2) depletion of glutathione (GSH) in tumor cells; and (3) initiation of Mn^2+^ medicated Fenton-like reaction to generate ROS, all of which contributed to the enhanced anti-tumor efficacy of the nanomaterial [51]. Nonlinear absorption of two relatively low-energy photons of NIR light is associated with the emission of high-energy visible light that can sensitize oxygen to produce cytotoxic ROS including singlet oxygen which can kill cancer cells [52]. Lu et al., [53] attributed cell death caused by mitochondria-mediated apoptosis of MCF-7 breast cancer cells exposed to low concentration (1.6 μg/mL) of Ir(tiq)_2_ppy NPs under white light irradiation at quite low intensity (5 mW cm^−2^) to excessive generation of ROS under light irradiation. Hou et al., [54] developed a multifunctional nanoplatform to enhance PDT efficiency by increasing the generation of ROS in tumor cells through Fenton reaction and reducing the distance between ROS and target site by targeting the mitochondria.

The results of MRI demonstrated a concentration dependent enhancement of signal intensity with increasing concentration of NDG-Mn_3_O_4_ nanocomposites (Figure 8). Although MRI is the best imaging technique for detecting soft tissue, the long relaxation time of water protons led to weak differences between tissues, resulting in poor image depiction between normal and malignant tissue [55]. However, the use of contrast agents (CAs) significantly enhanced the quality of MRI images and therefore the sensitivity of MRI-based clinical diagnosis [56]. Gadolinium (Gd)-based T1 contrast agents have been commonly used in clinical practice [57], however, they have the drawbacks of short blood circulation time and nephrotoxicity [58]. In recent years, due to their good biocompatibility, relatively high magnetization spin and rapid water proton exchange rate, manganese oxide nanoparticles have been developed as T1 contrast agents that have shown significant potential for detection and diagnosis of cancer [9,28,29].

## 4. Materials and Methods

### 4.1. Materials

Manganese (II) acetylacetonate, oleylamine, graphite powder, sodium nitrate, suphuric acid, ammonium hydroxide, hydrazine hydrate, potassium permanganate, hydrogen peroxide and other oranic solvents were purched from Sigma-Aldrich, St. Louis, MO 68178, USA. 

### 4.2. Preparation of Mn_3_O_4_ Nanoparticles

Manganese (II) acetylacetonate was dissolved in oleylamine (molar ratio of manganese (II) acetylacetonate: oleylamine = 1:25) and the mixture was heated at 160 °C for 10 h under a nitrogen cover. The resulting product was cooled to room temperature to form a brownish suspension, which was centrifuged at 9000 rpm for 15 min and the supernatant was removed to obtain a brown residue. The precipitate was washed multiple times with ethanol to acquire pure Mn_3_O_4_ nanoparticles, which were dried under vacuum before use [34].

### 4.3. Preparation of Nitrogen-Doped Graphene Oxide (NDG)

Initially, graphite oxide (GO) was synthesized from graphite powder using a modified Hummers method [59,60]. Briefly, graphite powder (0.5 g) and NaNO_3_ (0.5 g) were added to 23 mL of H_2_SO_4_ and the mixture was stirred for 10 min in an ice bath. Subsequently, KMnO_4_ (3 g) was slowly added and after proper mixing, the ice bath was replaced with water bath (35 °C) for 1 h, resulting in the formation of a thick paste. Thereafter, 40 mL of deionized water was added, and the mixture was stirred for 30 min at 90 °C. Finally, 100 mL of deionized water was added, followed by the slow addition of 3 mL of H_2_O_2_. The mixture was allowed to cool, filtered and washed with deionized water. The resulting thick brown paste was dispersed in water and centrifuged at 1000 rpm for 2 min. This step was repeated 4–5 times, until all unsettled particles were removed. The resultant paste was dispersed in water with mild sonication to obtain a suspension of graphene oxide (GO). For nitrogen doping, the resulting suspension was taken in a round bottom flask, to which 4 mL of NH_4_OH and 4 mL hydrazine hydrate were added simultaneously. The mixture was stirred for a few minutes, and the flask (equipped with cooling condenser) was put in a water bath controlled at 90 °C for 3 h. The product was collected after been filtered through micropore filters (Whatman filter paper, pore size-20 μm, W&R Balston Limited, Maidstone, Kent, UK), washed by deionized water and freeze-dried.

### 4.4. Preparation of Nanocomposites of NDG and Mn_3_O_4_ (NDG-Mn_3_O_4_) 

Equal amounts of Mn_3_O_4_ nanoparticles and NDG were milled using a Fritsch Pulverisette P7 planetary ball mill (Idar-Oberstein, Germany). The nanomaterials powder and stainless steel balls (5 mm diameter) with the ball to powder weight ratio of 1:1 were introduced into the stainless steel container. The milling of the powder was performed for 16 h, with intermittent pausing of milling process at regular intervals.

### 4.5. Characterization of Nanoparticles

The synthesized nanoparticles were characterized for size and physicochemical properties using high resolution transmission electron microscopy (JSM-7610F, JEOL, Tokyo, Japan), X-ray diffraction analysis (D2 Phaser X-ray diffractometer, Bruker, Ettlingen, Germany) and FT-IR spectroscopy (Perkin Elmer 1000 FT-IR spectrometer, Waltham, MA, USA). 

### 4.6. Cell Viability Analysis

We used the 3-(4,5-dimethylthiazol-2-y1)-2,5-diphenyltetrazolium bromide (MTT) method for testing the cytotoxicity of Mn_3_O_4_ and NDG-Mn_3_O_4_ nanoparticles. A549 lung cancer cells were seeded into 96-well plate (4 × 10^4^ cells per well) in RPMI and incubated at 37 °C for 4 h in a 5% CO_2_ incubator. Different concentrations (6.25–100 µg/mL) of Mn_3_O_4_ and NDG-Mn_3_O_4_ nanoparticles were added to the 96-well plate. Phosphate buffer saline (PBS) was used as a control whereas triton-X100 was used as negative control. The cells were treated with a 670 nm laser irradiation at 0.1 W/cm^2^ for 5 min and further incubated for 24 h. Aqueous solution of MTT (50 µL) was added to each well in the 96-well plate 4 h before the termination of 24 h incubation. The upper layer of the solution was discarded. The MTT solubilization solution, DMSO (100 µL) was added to each well to dissolve the formazan crystals by pipette stirring and then observed the absorbance at 590 nm, which was converted to cell viability using the following equation [34].

Cell viability (%) = (absorbance of sample cells/absorbance of control cells) × 100

### 4.7. In-Vitro Photodynamic Therapy

We used fluorescence microscopy for morphological analysis of cancer cells following treatment with nanoparticles and laser irradiation. Fluorescein diacetate (FDA) and propidium iodide (PI) were used to visualize the live and dead cells, respectively. A549 cells (2 × 10^4^ cells per well) were seeded in a 24 well plate and incubated at 37 °C for 24 h in an atmosphere of 5% CO_2_. Mn_3_O_4_ and NDG-Mn_3_O_4_ nanoparticles (50 µg/mL) were added to the wells and the plate was incubated for 4 h. After incubation, the cells were irradiated for 5 min with a 670 nm laser, followed by another incubation for 24 h. Both the dyes were added to wells and the plate was incubated for 5 min. Then, the cells were washed three times with PBS to remove excess dyes, and the fluorescence images were acquired by fluorescence microscope with 490 nm excitation and 525 nm emission wavelengths.

### 4.8. Analysis of Singlet Oxygen Generation

We used 1,3-diphenylisobenzofuran (DBPF) to detect singlet oxygen (^1^O_2_) generation by NDG-Mn_3_O_4_ nanocomposites under 670 nm laser irradiation (0.1 W/cm^2^). Fifty microliters of ethanolic solution of DPBF (1 mg/mL) were added to the nanocomposites solution under stirring and irradiated with laser for different time points. The absorbance of solution was measured by UV-Visible spectrophotometer. The decrease in absorbance at 426 nm indicated the degradation of DPBF in presence of ^1^O_2_ which was generated by laser-induced activation of NDG-Mn_3_O_4_ nanocomposites. 

### 4.9. MRI Relaxivity Analysis

A series of aqueous suspensions of NDG-Mn_3_O_4_ nanoparticles (with Mn concentration from 0 to 1 mM) were prepared and imaged in 0.2 mL Eppendorf tubes using a 3T clinical MRI instrument (GE Signa Excite Twin-Speed, GE Healthcare, Milwaukee, WI, USA). The specific relaxivity (r_1_) was calculated from linear curve generated from concentration of NDG-Mn_3_O_4_ nanocomposites versus 1/T_1_ (s^−1^).

### 4.10. Statistics

The data were analyzed by one-way analysis of variance (ANOVA) followed by Dunnett’s test. *p* values < 0.05 were considered as statistically significant. 

## 5. Conclusions

The NDG-Mn_3_O_4_ nanocomposites did not cause any cytotoxicity under physiological conditions. However, they produced significant and dose-dependent cytotoxicity in cancer cells after 670 nm laser irradiation. The PDT potential of NDG-Mn_3_O_4_ nanocomposites was attributed to excessive generation of ROS in exposed cells. NDG-Mn_3_O_4_ nanocomposites also exhibited concentration-dependent MRI contrast property suggesting their possible application for cancer imaging. Further studies are warranted to test the theranostic potential of NDG-Mn_3_O_4_ nanocomposites in animal models of cancer. 

## Data Availability

Data contained within the article.
